# Association of Cortical Vein Filling with Clot Location and Clinical Outcomes in Acute Ischaemic Stroke Patients

**DOI:** 10.1038/srep38525

**Published:** 2016-12-05

**Authors:** Sonu Bhaskar, Andrew Bivard, Peter Stanwell, John R. Attia, Mark Parsons, Michael Nilsson, Christopher Levi

**Affiliations:** 1 Department of Neurology, John Hunter Hospital, University of Newcastle, Australia; 2 Centre for Translational Neuroscience and Mental Health, School of Health Sciences and Hunter Medical Research Institute, University of Newcastle, Australia; 3 Centre for Clinical Epidemiology & Biostatistics, Hunter Medical Research Institute, University of Newcastle, Australia; 4 Centre for Brain Repair and Rehabilitation, Institute of Neuroscience and Physiology, Sahlgrenska University Hospital, University of Gothenburg, Gothenburg, Sweden

## Abstract

Delay in cortical vein filling during the late-venous phase (delayed-LCVF) is characterized by opacification of cerebral veins despite contrast clearance from contralateral veins on dynamic computed tomography angiography (dCTA) in acute ischemic stroke (AIS) patients. The aim of the study was to investigate the associations of delayed-LCVF with clot location, reperfusion status at 24 hours, and 90-days functional outcome in AIS patients who received reperfusion therapy. A prospective cohort of AIS patients treated with intravenous thrombolysis was studied. Groupwise comparison, univariate, and multivariate regression analyses were used to study the association of delayed-LCVF with clot location and clinical outcomes. Of 93 patients (mean age = 72 ± 12 years) with hemispheric AIS included in the study, 46 (49%) demonstrated delayed-LCVF. Patients with delayed-LCVF demonstrated a significantly higher proportion of proximal occlusion (72% vs 13%, P =< 0.0001), and poor reperfusion at 24 hours (41% vs 11%, P = 0.001). The proportion of poor functional outcome at 90 days was not significantly different (22/56 (48%) vs 17/61 (36%), P = 0.297). The appearance of delayed-LCVF on baseline dCTA may be a surrogate for large vessel occlusion, and an early marker for poor 24-hour angiographic reperfusion.

Identification of patients who are most likely to benefit from reperfusion therapy using clinical and imaging markers is important in the quest for a more tailored approach to treatment in acute ischemic stroke (AIS). We recently reported a novel CT angiographic (CTA) finding on the presence of delayed cortical vein filling in late-venous phase in AIS patients^1^. Delayed late-phase cortical vein filling (delayed-LCVF) is characterised by late-venous phase opacification of cortical veins despite contrast clearance from contralateral cortical veins on four-dimensional (4D) dynamic time-resolved CTA (dCTA). We found that delayed-LCVF is independently associated with poor baseline collateral status. Currently, assessment of arterial leptomeningeal collateral status is indirect and performed qualitatively through visual examination of the extent and rate of backfilling of pial arteries that are fed by collateral vessels. Therefore, delayed-LCVF, a more ‘direct’ and reproducible measure, may prove to be very helpful to assess collateral status. There is growing interest in the role of leptomeningeal collaterals in AIS^1^,^2^,^3^,^4^,^5^,^6^,^7^,^8^. The presence of baseline arterial collaterals is emerging as an important parameter in the evaluation and treatment of cerebral ischemia^9^, and is linked to infarct core volume^10^, and functional outcomes^6^,^11^,^12^. Good collaterals are associated with good clinical outcomes; conversely, poor collaterals are linked to infarct growth^3^. Studies on the role of cortical veins in stroke pathophysiology and prognosis are limited^1^,^2^,^12^,^13^,^14^,^15^,^16^,^17^.

Location and the volume of the thrombus are also important factors in prognostication of AIS^18^,^19^,^20^,^21^,^22^,^23^,^24^,^25^,^26^,^27^. Proximal, high volume clots have poor clinical outcomes, while, low-volume, distal thrombus is associated with good clinical outcomes. Moreover, the size of thrombus and the anatomical differentiation between a proximal and a distal occlusion also influences the effectiveness of intra-venous thrombolysis (IVT) with recombinant tissue plasminogen activator (rtPA)^18^,^19^,^20^,^21^,^22^,^23^,^24^,^25^,^26^. IVT is more efficient in the dissolution of distal clots in comparison to proximal ones. Therefore, further studies on the association of thrombus location with delayed-LCVF, in AIS patients who received IVT, are important.

In this study, we prospectively studied the association of late stage cortical vein drainage in a group of AIS patients treated with IVT, with tissue at risk, clot location, and clinical outcome. The specific objectives of the study were:

1. To study the association of delayed-LCVF with ischemic infarct core and tissue at risk.
2. To study the association of delayed-LCVF with clot location.
3. To investigate if delayed-LCVF is associated with clinical outcomes including reperfusion status at 24 hours, and 90-day functional outcome.

We hypothesise that patients with delayed-LCVF will have smaller penumbra, higher infarct core volumes, and worse outcomes at 90 days. We also hypothesise that the AIS patients with proximal occlusion (M1 proximal (M1P) or ICA) will demonstrate a higher proportion of delayed-LCVF patterns in comparison to the patients with distal occlusion (M1 distal (M1D) or M2 or M3). We discuss the implications of the location of the clot in evaluating the clinical outcomes post IVT in the AIS cohort.

## Results

### Case Presentation

**Figure 1:**
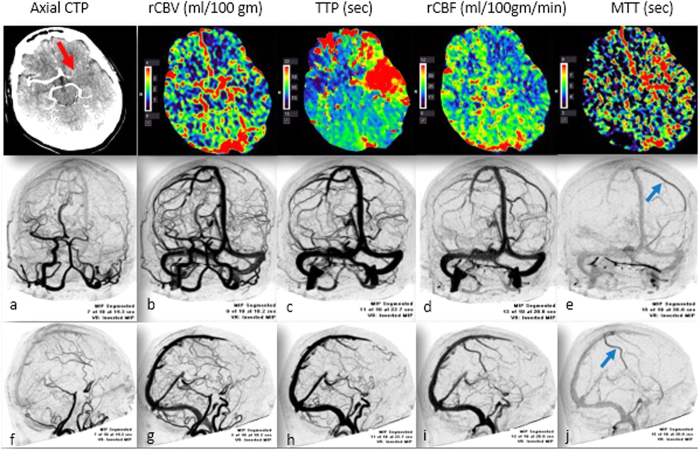
Case Study 1. Acute stroke imaging. Top Panel: Axial CTP demonstrated the presence of an occlusion in the left MCA. The red arrow points to the presence of a left MCA occlusion. CTP (coronal 5 mm average registered) shows the presence of penumbra and demonstrated region of reduced cerebral blood flow (CBF) and blood volume (CBV), as well as increased mean transit time (MTT) and time to peak (TTP) in the left MCA cortex. 2D MIP spiral dynamic CT angiography (CTA) images formatted in coronal (middle panel) (**a**–**e**), and sagittal (bottom panel) planes (**f**–**j**); CTA Right-Left view and CTA posteroanterior view. The Early phase is characterized by early filling of venous sinuses (**a**), followed by mid-venous phase (**b**–**d**). Late venous phase is depicted in (**e**) (blue-arrow). The presence of left-sided late-venous phase opacification of cortical veins (blue arrow) on left side despite contrast clearance from contralateral (right side) cortical veins can be seen on dynamic CTA image, (**e**,**j**). *Background: A 72-year-old female with a history of hypertension, diabetes, hyperlipidaemia, and atrial fibrillation presented with right-sided facial droop and right-sided hemiparesis with NIHSS score of 21 on admission. Acute CTP demonstrated acute right middle cerebral artery (MCA) ischemia with evidence of a large penumbra. The patient demonstrated delayed-LCVF on baseline dCTA. The patient received intravenous rt-PA therapy at 90* *minutes’ post stroke onset.*

**Figure 2:**
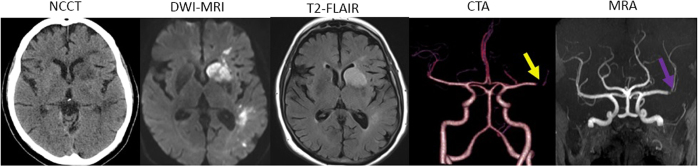
Case Study 1. Follow-up imaging at 24 hours. Non-contrast CT (NCCT) at 24 hours shows low attenuation within the head of the left caudate nucleus, anterior limb of the left internal capsule and lentiform nucleus, as well as patchy low attenuation in the white matter in the corona radiata and temporal lobe, consistent with the known left middle cerebral artery (MCA) infarct. No evidence of haemorrhagic transformation of areas of parenchymal abnormality was seen. DWI-MRI showed patchy infarcts in left MCA territory. T2-FLAIR also confirmed the presence of patchy infarcts in the left hemisphere. Magnetic resonance angiography (MRA) shows that the left MCA has reanalysed however, there remains some poor flow into the branches with the suggestion of some stenosis at the bifurcation. 24 hour CTA showed evidence of partial recanalization. Poor flow at the bifurcation possibly due to stenosis. *Background: Despite thrombolysis, the patient showed only partial recanalization/reperfusion. On 24* *hour CTA, poor flow into the branches was observed suggestive of stenosis at the bifurcation despite recanalization. There is evidence of infarction involving the basal ganglia and some patchy changes in the left MCA territory. Long-term prognosis of the patient was poor (mRS* = *4 at 90 days).*

A case study showing the acute CTP and dCTA at baseline is shown in Fig. 1. Figure 2 depicts the follow-up CT & MR imaging findings at 24 hours.

### Baseline characteristics

**Figure 3:**
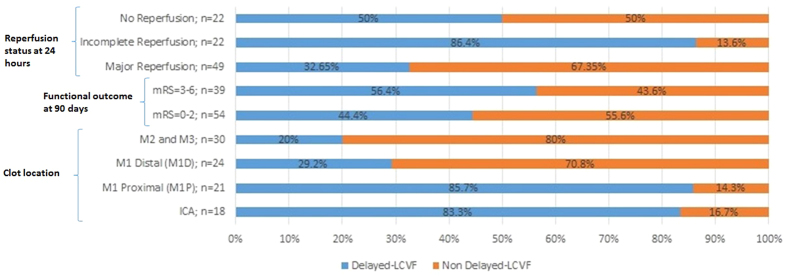
Clinical outcome (reperfusion status at 24 hours, and 3-month modified Rankin score (mRS)) and clot location for presence and absence of delayed-LCVF. Good clinical outcome was defined as mRS 0–2 at 3 months.

Of the 154 patients, 93 (60.4%) patients (mean age = 71.6 ± 12.4 years; the number of females = 49 (52.7%)) with acute anterior circulation vessel occlusion who received IVT met the inclusion criteria (Table 1). Figure 3 shows the distribution of patients for different clot locations, reperfusion status at 24 hours and functional outcome at 90 days. Out of 93 patients with hemispheric ischaemic stroke included in the study, 46 (49.5%) patients showed delayed-LCVF. The median NIHSS score at admission and 24 hours were 14 (IQR = 8) and 7 (IQR = 9), respectively. Fifty-four (58%) patients demonstrated good functional outcome (mRS 0–2) at 90 days and 52.7% of patients demonstrated major reperfusion at 24 hours. The average OTT for this AIS cohort was 162.6 ( ± 83.9) minutes. The summary of clinical outcomes for all patients is shown in Table 2.

### Delayed-LCVF vs No delayed-LCVF

There were no significant differences between the age, sex, NIHSS at admission and at 24 hours, and other clinical risk factors among patients with, and without, delayed LCVF. Patients with delayed-LCVF demonstrated a significant association with poor baseline collaterals (85% vs 21%, P =< 0.001; OR = 20.6; 95% CI = [7, 60]; P=< 0.001), and longer time to peak of maximum arterial enhancement (TPME) (median TPME (in seconds) = 8 vs 6, P =< 0.001; OR = 3.3; 95% CI = [2, 5.4]; P =< 0.001) (Table 1). There were no significant differences in onset to treatment time (OTT) between the delayed vs non-delayed LCVF groups (P = 0.215). We also observed no significant association of OTT with 90 days’ functional outcome (P = 0.2090) which is in agreement with previous study^27^.

Occlusion of the internal carotid artery (ICA) (32.6% vs 6.4%, P = 0.002), and M1 proximal (M1P) (29% vs 6.4%, P =< 0.001) was significantly higher in the delayed-LCVF group in comparison to non-delayed-LCVF. Conversely, distal M1 (M1D) (15% vs 36%, P = 0.03), and M2 and/or M3 (13% vs 51%, P =< 0.001) occlusions were significantly lower in delayed-LCVF group. When pooled, proximal occlusion (ICA and/or M1P) was commonly seen in delayed-LCVF (72% vs 13%, P =< 0.001) group, and conversely, occurrence of distal occlusion (M1D and/or M2 and/or M3) was significantly lower in delayed-LCVF group (28% vs 87%, P =< 0.001), versus the non-delayed-LCVF group.

### Association of delayed-LCVF with infarct core and tissue at risk

Acute core volumes were not significantly different between delayed-LCVF vs non-delayed LCVF groups (18.85 mL vs 13.15 mL, P = 0.731) (Table 1). Univariate analysis revealed no significant association of the acute core volume and presence of delayed-LCVF (OR = 1; 95% CI = [0.99, 1.02]; P = 0.494). In terms of penumbra volume, no significant difference was found between the two groups, delayed-LCVF vs non-delayed LCVF (61.05 mL vs 64.45 mL, P = 0.574). Moreover, delayed-LCVF was not associated with penumbra volume (OR = 1.01; 95% CI = [0.99, 1.01]; P = 0.732). No significant association was found between the delayed-LCVF and 24-hour core volume (18 mL vs 12.5 mL, P = 0.37; OR = 1, 95% CI = [1, 1.01], P = 0.247) (Table 2). Moreover, delayed-LCVF was also not associated with penumbral salvage (24 mL vs 43 mL, P = 0.31; OR = 1, 95% CI = [0.99, 1], P = 0.272).

### Associations with clot location

Results of univariate logistic regression analysis for association with proximal clot is shown in Supplementary Table 2. Independent variables (with P ≤ 0.1) and other important covariates (NIHSS at admission) were used for stepwise backward multivariate logistic regression analysis to study the association with incidence of the proximal clot (see Table 3; Model 3A). Finally, TPME, dyslipidemia, and delayed-LCVF were retained in the final multivariate logistic regression model and treated as potential confounders (Table 3; Model 3C). Higher rates of delayed-LCVF (OR = 106.62; 95% CI = [15, 756]; P =< 0.0001) and dyslipidemia (OR = 5.8; 95% OR = [1.6, 21]; P = 0.007) were positively associated with incidence of proximal clot. Interestingly, each unit increase in TPME was negatively associated with presence of proximal clot (OR = 0.56; 95% CI = [0.35, 0.9]; P = 0.02). The model showed good discrimination ability with an area under the receiver operating characteristic (ROC) curve of 0.89 (sensitivity = 82%, specificity = 81.5%). We also compared the reduced models with and without the inclusion of delayed-LCVF (Model 3C (reduced model with delayed-LCVF) vs Model 3B (reduced model without delayed-LCVF)). We found that the addition of delayed-LCVF significantly increased the discrimination accuracy of the model (BIC_Model3C_ vs BIC_Model3B_ = 95 vs 131; ROC_Model3C_ vs ROC_Model3B_ = 0.89 vs 0.66; Sensitivity_Model3C_ vs Sensitivity_Model3B_ = 82% vs 36%; Specificity_Model3C_ vs Specificity_Model3B_ = 81.5% vs 81.5%; PPV_Model3C_ vs PPV_Model3B_ = 76.19% vs 58.3%).

#### Delayed-LCVF association with reperfusion status at 24 hours, and functional outcome at 90 days

Patients with delayed-LCVF demonstrated a significantly higher proportion of poor angiographic reperfusion at 24 hours (68% vs 31.8%, P = 0.001) (Table 2). Moreover, bivariate logistic regression analysis also revealed a significant association of delayed-LCVF with overall angiographic reperfusion status at 24 hours (P =< 0.001). Delayed-LCVF was positively associated with poor reperfusion at 24 hours (OR = 4.4; 95% CI = [1.8, 10.6]; P = 0.001). Independent variables (with P ≤ 0.1; age, NIHSS at admission, acute core volume, collateral status, delayed-LCVF, clot location and hypertension) (see Supplementary Table 3) were used for stepwise backward multivariate logistic regression analysis to study the association with poor angiographic reperfusion (see Table 4; Model 4 A). In the reduced model (Table 4; Model 4 C), delayed-LCVF, clot location, and baseline core volume were retained. Higher rates of delayed-LCVF (OR = 3.7; 95% CI = [1.2, 11.28]; P = 0.021), and increasing acute core volume (OR = 1.02; 95% OR = [1, 1.04]; P = 0.021) were significantly associated with poor angiographic reperfusion at 24 hours. Comparison between the reduced models with and without inclusion of delayed-LCVF (Model 4C (reduced model with delayed-LCVF) vs Model 4B (reduced model without delayed-LCVF)) revealed that the addition of delayed-LCVF to the model revealed no added advantage on discriminative accuracy: BIC (BIC_Model4C_ vs BIC_Model4B_ = 127 vs 128), sensitivity (Sensitivity_Model4C_ vs Sensitivity_Model4B_ = 70.45% vs 70.45%), or discrimination accuracy (ROC_Model4C_ vs ROC_Model4B_ = 0.74 vs 0.70; Specificity_Model4C_ vs Specificity_Model4B_ = 68.75% vs 64.58%; PPV_Model4C_ vs PPV_Model4B_ = 67.4% vs 64.58%) (Table 4). In the model without delayed-LCVF (Model 4B), proximal clot (OR = 2.9; 95% CI = [1.2, 7.17]; P = 0.017) and acute core volume (OR = 1.02; 95% CI = [1, 1.04]; P = 0.015) were significantly associated with poor angiographic reperfusion status at 24 hours.

Delayed-LCVF was not significantly associated with penumbral salvage (Median Penumbral Salvage, in mL = 24.15 vs 43.45, P = 0.31; median penumbral salvage, in percentage = 52.9% vs 84.2%, P = 0.1) or functional outcome at 90 days (47.8% vs 36%, P = 0.297) (Table 2). To study the association with functional outcome at 90 days using backward stepwise multivariate regression analyses, independent variables (with P ≤ 0.1; NIHSS at admission, baseline core volume, penumbra, dyslipidaemia, and reperfusion status at 24 hours (see Supplementary Table 1) and other important covariates (clot location, delayed-LCVF) were included in the final multivariate regression model (Table 5; Model 5A). In the reduced multivariate regression model (Model 5C), delayed-LCVF was not significantly associated with functional outcome at 90 days, when adjusted for NIHSS at admission, acute core volume, clot location, and 24-hour reperfusion status (Table 3). Increasing acute core volume and reperfusion status at 24 hours were significantly associated with poor functional outcome at 90 days. Comparison of model characteristics between the two models, with and without delayed-LCVF, revealed addition of delayed-LCVF significantly improved the discriminative accuracy: BIC (BIC_Model5C_ vs BIC_Model5B_ = 92.5 vs 116), sensitivity (Sensitivity_Model5C_ vs Sensitivity_Model5B_ = 82% vs 69%), or discrimination accuracy (ROC_Model5C_ vs ROC_Model5B_ = 0.93 vs 0.80; Specificity_Model5C_ vs Specificity_Model5B_ = 88.7% vs 79.25%; PPV_Model4C_ vs PPV_Model4B_ = 84.21% vs 71%) (Table 5).

## Discussion

In this study, we sought to investigate associations of novel cortical vein filling pattern, observed during the late venous phase on time-resolved dCTA, with the acute core, tissue at risk, clot location, reperfusion status at 24 hours, and long-term functional outcomes in a cohort of AIS patients treated with IVT. In the current study, we found that the patients with proximal (thrombus in ICA and/or M1P) occlusion are at significantly higher risk of showing delayed-LCVF. We also noted a strong association of delayed-LCVF with poor reperfusion status at 24 hours. We could not demonstrate that the impact of delayed-LCVF on these acute outcomes translated into poor functional outcomes at 90 days, but this may be due to our small sample size and the fact that functional outcome is quite distal in the causal chain we are investigating. The inclusion of delayed-LCVF to the multivariate model significantly improved the predictive accuracy of poor functional outcome. We found no association of delayed-LCVF with ischemic infarct core or tissue at risk. Previously, we reported the presence of delayed cortical vein filling pattern in late-venous phase on dCTA in a cohort of AIS patients, where delayed-LCVF was found to be independently associated with poor baseline arterial collaterals and delay in maximised collateral enhancement^1^. There are limited studies on the role of cortical veins in stroke pathophysiology, and their associations with thrombus location and clinical outcome^13^,^17^,^28^,^29^,^30^,^31^.

Our findings indicate that delayed-LCVF was significantly more common in patients with M1P and/or ICA occlusion. Delayed-LCVF showed significant improvement in discriminative accuracy when it was added to the multivariate regression model. As such, the appearance of delayed-LCVF on dCTA is a surrogate for proximal large vessel occlusion which is known to be associated with poor outcome. Previous studies have reported that AIS patients with thrombus in M1D, M2, and M3 segments are more likely to undergo recanalization than those with M1P and ICA occlusions^18^,^27^. Large vessel occlusions are less likely to be recanalised after IVT and are more likely to have poor clinical outcomes. The fact that delayed-LCVF is strongly associated with proximal thrombus may be used as an important parameter towards stroke prognostication and selection of patients for IVT. We postulate that proximal large vessel occlusions may lead to delayed-late cortical vein filling.

In this study, we found that patients who showed delayed-LCVF on baseline dCTA demonstrated the significantly higher rate of poor angiographic reperfusion at 24 hours. This is clinically relevant suggesting that patients with the delayed-LCVF pattern on baseline dCTA will be poorly reperfused despite IVT. However, the addition of delayed-LCVF to multivariate regression model didn’t improve the predictive accuracy of the model over and above clot location. Our results show that delayed-LCVF is a statistically significant prognostic indicator of early angiographic reperfusion (at 24 hours). From the current literature, we know that reperfusion status is a significant predictor of long-term (90 days) outcome^32^,^33^. In a multivariate logistic regression analysis, delayed-LCVF was not a significant covariate for predicting 90 days’ functional outcome. Although, our point estimate supports an effect on 90 days mRS (OR = 1.6), we do not have sufficient power to demonstrate this at a statistically significant level. We hypothesize that patients with the delayed-LCVF pattern on baseline dCTA may show an unfavourable trajectory. Interestingly, the addition of delayed-LCVF significantly improved the predictive accuracy of functional outcome at 90 days. The presence of delayed-LCVF may aid in identifying patients at risk of 24-hour poor angiographic reperfusion. Other studies have also shown significant association of cerebral-venous flow with prognosis in stroke in both animals and humans^13^,^17^,^34^. Interestingly, animal studies focussing primarily on early and mid-venous phase have shown that the presence of cortical vein filling after ischaemic stroke was associated with decreased severity of hemiparesis and lower infarct volumes. This led to the hypothesis that the cortical venous flow may produce favourable outcomes as it would be more commonly prevalent in strokes with good collaterals^34^. Another study on humans also found that the cortical venous drainage was associated with good clinical outcomes^17^.

Imaging biomarkers towards the identification of patients who might benefit from early reperfusion therapy and guiding early intervention options to limit or even freeze infarct progression is crucial for strategies in acute stroke treatment^35^,^36^. The advent of cutting-edge next generation 320-detector row 640-slice multi-detector CT (MDCT) scanners have facilitated the acquisition of whole-brain, sub-second, and volumetric acquisition of 4D-dCTA data^1^,^37^,^38^. CTP/CTA is not an invasive procedure compared to digital subtraction angiography (DSA), and is routinely obtained during clinical care of stroke patients^8^,^32^,^33^,^39^, has proven to be of added clinical utility in the early evaluation of stroke, facilitating precise localization of site of occlusion^40^, and identification of hypoperfused territory at risk of infarction^41^. The CTP was acquired simultaneously with the CTA with the use of same contrast bolus^42^. Dynamic CTA allows evaluation of intracranial vasculature and visualisation of contrast flow from its arterial to venous phases. Using appropriate reconstructions of dCTA using MIP algorithm, we investigated various stages of venous drainage and downstream venous dynamics; including the assessment of delayed-LCVF appearance in late venous phase. Assessment of impaired cortical venous drainage may provide valuable information over and above arterial collateral assessment, and the presence of delayed-LCVF could have a role in making informed decisions on patient management and prognosis.

We understand that our study has several limitations, including small sample size and the variability in the cortical venous structures. Since the publication of MR CLEAN, REVASCAT^43^,^44^, EXTEND-IA, ESCAPE, and SWIFT PRIME, the standard practice now includes intravenous thrombolysis when possible, complementing thrombectomy^43^,^44^,^45^,^46^. However, endovascular treatment was not available at our centre at the time of the study. We tried to account for small sample size by using the Wilcoxon-Rank test, which would be conservative in this case. We acknowledge that additional occlusions distal to the M1 segment and variations in the prominences of M2, M3, and M4 trunks may have an impact on the assessment of delay in maximised enhancement. Moreover, it may also be influenced by the differences in the filling time of collaterals in different areas of the MCA territory.

To conclude, in this study, we sought to study the associations of delayed-LCVF with core volume, tissue at risk, clot location, and clinical outcome (vis a vis reperfusion at 24 hours, functional outcome (in terms of modified Rankin score (mRS)) at 90 days) in a prospective cohort of AIS patients who received intravenous thrombolytic therapy. Endovascular procedures or mechanical thrombectomy was not available at our centre at the time of the study. Delayed-LCVF patterns were more commonly seen in proximal thrombus occlusion in M1P and/or ICA. Based on these findings, the appearance of delayed-LCVF on dCTA can be used as a surrogate for proximal thrombus or large vessel occlusion. It may also be useful in identifying patients at risk of poor angiographic reperfusion at 24 hours. Moreover, given the propensity of proximal thrombus towards poor clinical outcome after IVT^47^, and significant association of delayed-LCVF with both proximal thrombus and poor reperfusion at 24 hours, we postulate that the AIS patients with delayed-LCVF may progress unfavourably, and therefore alternate revascularisation strategies may be considered. We also found that addition of delayed-LCVF significantly improves predictive accuracy of 90 days’ functional outcome. However, in the present cohort, we acknowledge that delayed-LCVF was not found to be a determinant factor in predicting functional outcome at 90 days. In light of the paucity of literature on the cerebral venous system and their role in stroke prognostication, we believe this study may be of clinical relevance towards understanding the role of cerebro-venous system, in particular, cortical vein, in the prognosis of AIS patients. We propose that delayed-LCVF is a marker that will allow clinicians to extract more prognostic information from imaging that is already routinely acquired. Also, in combination with NCCT, CTA/CTP can be rapidly obtained with minimal delay in treatment, and is widely available in emergency departments, and is well tolerated^41^. Dynamic CTA is a promising technique for the dynamic assessment of the cerebral vasculature. We caution that these results must be understood as preliminary and within the context of the study design. Further prospective studies are recommended to study the role of cortical veins in stroke prognostication.

## Materials and Methods

### Study design and patient selection

Consecutive acute ischaemic stroke patients admitted to the comprehensive stroke unit, Department of Neurology at our academic medical centre were prospectively studied provided they satisfied the following inclusion criteria: (a) aged 18 and above years, (b) acute anterior circulation vessel occlusion followed by IVT, (c) hemispheric stroke, and (d) dCTA data available at baseline and 24 hours. Patients without identifiable thrombus on the baseline dCTA were excluded. Patients received 0.9 mg/kg intravenous recombinant tissue plasminogen activator (rtPA). Baseline clinical characteristics included age, sex, and clinical risk factors (hypertension, diabetes, dyslipidaemia, history of smoking (past/present), atrial fibrillation (AF), depression and history of stroke and/or transient ischemic attack (TIA)). Clinical data were procured from the patient records. National Institutes of Health Stroke Scale (NIHSS) scores at the time of initiation of the rtPA and at 24 hours were obtained. The time delay between stroke onset and administration of tPA (time to tPA) or onset to treatment (OTT) was also recorded. Management of patients was in accordance with local guidelines and as per the discretion of the treating stroke physician. This study was approved by the Hunter New England Human Research Ethics Committee (HNEHREC, Newcastle, NSW) in accordance with the National Statement on Ethical Conduct in Human Research 2007. All methods were carried out in accordance with the approved guidelines. Informed consent was obtained from the patient in accordance with the Declaration of Helsinki.

All the patients underwent non-contrast CT (NCCT), CT Perfusion (CTP) and CT angiography (CTA) at baseline and follow-up (24 h) NCCT, CTA, and magnetic resonance imaging (MRI), following our routine stroke imaging protocol^1^. Volumes of the acute perfusion lesion (relative delay time (DT) ≥ 3 seconds) and acute infarct core (relative CBF ≤ 30%) were calculated using previously validated thresholds^8^,^48^. Penumbra volume was defined as the volume of the perfusion lesion (DT threshold ≥ 3 seconds) minus the volume of the infarct core (relative CBF threshold < 30% within the DT ≥ 3 sec lesion). The threshold of DT ≥ 3 seconds was based on previous studies^48^,^49^.

Maximum intensity projection (MIP) and multiplanar reformat (MPR) reconstructions in coronal and sagittal planes of baseline axial CTA were obtained on the imaging workstation (Vitrea^®^ fX, Version 1.0, Vital Images, Minnetonka, MN, USA). These images were reviewed by consensus by two experienced readers (SB & CL). Three-dimensional volume rendering was applied to obtain the optimized spatial orientation and precise localisation of ischemic lesion. The determination of the location of the clot was based on the most proximal position of the occlusion. Clot location was divided into two groups: (a) proximal clot: any thrombus/occlusion in the M1 proximal (M1P) or ICA, and (b) distal clot: any thrombus/occlusion in M1 distal (M1D), M2 or M3^50^. The exact location of the thrombus or clot M1 proximal (M1P), M1 distal (M1D), M2, M3, or internal carotid artery (ICA)) was determined. ICA occlusion was determined based on the presence of a clot in ICA terminus. The M1 segment of the MCA was divided into two parts of equal length, namely the proximal (M1P) and the distal half (M1D)^27^. M1 MCA was defined as a vessel extending from the ICA bifurcation to the origin of the first major branch in the Sylvian sulcus. Delayed-LCVF was identified by late venous phase opacification of cortical veins despite contrast clearance from contralateral cortical veins on maximum intensity projection (MIP) images from dCTA. The time to peak of maximum arterial enhancement (TPME) was also recorded^1^. Collateral grading was done to assess the morphological status using dCTA data based on the degree of reconstitution of the MCA up to the distal end of its occlusion. Collateral grading was classified as ‘good’, ‘reduced’ or ‘poor’ using the Miteff scale^6^,^51^. Good collateral grading was assigned if the entire MCA distal to the occluded segment was reconstituted, i.e., if collaterals reconstituted vessels in the: (a) distal portion of the occluded vessel, or (b) proximal portion of the segment adjacent to the occluded vessel (e.g., if there was proximal M1 occlusion, the distal M1 or proximal M2 segments reconstituted)^6^,^51^. Collateral grading was assigned “poor” status if the reconstitution of the distal MCA was only partial, i.e., if collaterals reconstituted vessels in the: (a) distal portion of the segment adjacent to the occluded vessel, or (b) two segments distal to the occluded vessel, or (c) little or no significant reconstitution of the territory of the occluded vessel^6^,^51^.

### Outcome measures

The modified Rankin Scale (mRS) was used to assess clinical outcome in terms of functional status at 3 months. Patient outcomes were dichotomized into good (mRS 0–2) versus poor/bad (mRS 3–6). Angiographic assessment of the degree of reperfusion was done by an independent blinded reviewer on a repeat CTA acquired at 24 hours using modified thrombolysis in cerebral infarction (mTICI) score^52^. An mTICI grade of 2a, defined as tissue reperfusion in <50% of the occluded artery territory, was identified as partial reperfusion. Major reperfusion corresponded to tissue reperfusion in ≥50% of the occluded artery territory with grades of 2b or 3 on the mTICI scale. All patients with partial or nil angiographic reperfusion at 24 hours were lumped together into “poor reperfusion” category. Penumbral salvage was defined as the difference between the acute CTP lesion volume (PWI lesion) and the 24-hour DWI lesion volume^53^,^54^. We identified penumbral salvage in patients where 24-hour DWI lesion volume was smaller than the acute perfusion lesion volume. Percentage of penumbra salvaged was defined as (penumbral salvage volume/penumbra volume) × 100^55^.

### Statistical analysis

All the statistical analyses were performed using STATA (Version 10, 2001; College Station, TX, USA). Numerical values given are the means (±standard deviation) or medians (interquartile range) for age, core and penumbra volumes, mRS scores, NIHSS at admission, NIHSS at 24 hours, and change in NIHSS scores as appropriate. For ordinal or continuous data, Mann-Whitney (Wilcoxon rank-sum) test was used. Nominal data were analysed with the Pearson’s chi-squared (χ^2^) and the 2-tailed Fisher exact test. Groupwise comparison was made between the patients with and without delayed-LCVF. Group differences were considered significant at values of P < 0.05. To test the independent association of significant variables with delayed-LCVF, logistic regression models were fitted. Baseline infarct lesion volume and penumbra volume was dichotomized into small (≤25 mL) or large (>25 mL) pertinent to the findings on 25 mL threshold of core volume that accurately predicted the presence of penumbra (tissue at risk), response to thrombolysis, and excellent outcome^56^,^57^. A stepwise backwards multivariate logistic regression analyses was used to study the association of delayed-LCVF with clot location, 24-hour angiographic reperfusion status, and 90 days functional outcome. Independent variables with P ≤ 0.1 (on univariate regression) and other important clinical covariates were included in the multivariate logistic regression model. The sensitivity, specificity, positive predictive value (PPV), negative predictive value (NPV), and the overall rate of correct classification for the multivariate models were estimated. Finally, the receiver operating characteristic (ROC) curve for the regression model was plotted, and the area under the curve was computed to evaluate the discriminative ability.

## Additional Information

**How to cite this article**: Bhaskar, S. *et al*. Association of Cortical Vein Filling with Clot Location and Clinical Outcomes in Acute Ischaemic Stroke Patients. *Sci. Rep.* **6**, 38525; doi: 10.1038/srep38525 (2016).

**Publisher's note:** Springer Nature remains neutral with regard to jurisdictional claims in published maps and institutional affiliations.

## Supplementary Material

Supplementary Information

## Figures and Tables

**Table 1:** Demographic and baseline characteristics of all patients and by the late-phase cortical vein filling (delayed-LCVF).

Characteristics	All patients (n = 93)	Delayed-LCVF (n = 46)	Non-delayed-LCVF (n = 47)	P	OR (95% CI)	P > |z|
Age; Mean ± SD	71.6 ± 12.39	73.46 ± 11.06	69.79 ± 13.44	0.282	1.03 [0.99, 1.06]	0.157
Female; n (%)	49 (52.69)	26 (56.52)	23 (48.94)	0.535	1.36 [0.60, 3.07]	0.464
NIHSS at admission; Median [IQR]	14 [8]	13.5 [8]	14 [9]	0.408	1.02 [0.95, 1.09]	0.565
OTT (in mins); Mean ± SD	162.55 ± 83.87	172.76 ± 90.58	152.55 ± 76.4	0.215	1 [1, 1.01]	0.250
Acute Core Volume; Median [IQR]	13.55 [25.4]	18.85 [26.4]	13.15 [18.7]	0.731	1.0 [0.99, 1.02]	0.494
Large Lesion (Core > 25 mL)	27 (29.35)	16 (34.78)	11 (23.91)	0.360	1.70 [0.68, 4.21]	0.254
Small Lesion (Core ≤ 25 mL)	65 (70.65)	30 (65.22)	35 (76.09)	0.360	0.59 [0.24, 1.46]	0.254
Acute Penumbra Volume, ml; Median (IQR)	64.05 [77.65]	61.05 [78.3]	64.45 [65.7]	0.574	1.01 [0.99, 1.01]	0.732
Large penumbra (>25 mL)	67 (72.83)	31 (67.39)	36 (78.26)	0.349	0.57 [0.23, 1.46]	0.244
Small penumbra (≤25 mL)	25 (27.17)	15 (32.61)	10 (21.74)	0.349	1.74 [0.69, 4.43]	0.244
TPME; Median [IQR]	8 [2]	8 [2]	6 [2]	<0.001*	3.30 [2, 5.43]	<0.001*
Baseline Collateral Status				<0.001*		
Good Collaterals; n (%)	44 (47.31)	7 (15.22)	37 (78.72)	<0.001*	0.05 [0.07, 0.14]	<0.001*
Poor Collaterals; n (%)	49 (52.69)	39 (84.78)	10 (21.28)	<0.001*	20.6 [7.1, 59.83]	<0.001*
Clot location				<0.001*		<0.001*
Proximal thrombus (ICA + M1P)	39 (41.94)	33 (71.74)	6 (12.77)	<0.001*	17.3 [5.95, 50.59]	<0.0001*
Distal thrombus (M1D + M2 + M3)	54 (58.06)	13 (28.26)	41 (87.23)	<0.001*	1 (Ref)	
*ICA*	18 (19.35)	15 (32.61)	3 (6.38)	0.002*	7.1 [1.89, 26.6]	<0.004*
*M1 Proximal (M1P)*	21 (22.58)	18 (29.13)	3 (6.38)	<0.001*	9.43 [2.54, 34.97]	0.001*
*M1 Distal (M1D)*	24 (25.81)	7 (15.22)	17 (36.17)	0.032*	0.32 [0.12, 0.86]	0.024*
*M2 and M3*	30 (13.04)	6 (13.04)	24 (51.06)	<0.001*	0.14 [0.05, 0.40]	<0.0001*
Risk factors						
Hypertension	82 (88.17)	38 (82.61)	44 (93.62)	0.120	0.32 [0.08, 1.30]	0.113
Diabetes	27 (29.03)	14 (30.43)	13 (27.66)	0.822	1.14 [0.47, 2.8]	0.768
Dyslipidaemia	40 (43.01)	20 (43.48)	20 (42.55)	1.000	1.04 [0.46, 2.36]	0.928
Present Smoker	25 (26.88)	12 (26.09)	13 (27.66)	1.000	0.92 [0.37, 2.31]	0.864
Past Smoker	38 (40.86)	21 (45.65)	17 (36.17)	0.403	1.48 [0.65, 3.4]	0.353
AF	51 (54.84)	23 (50)	28 (59.57)	0.408	0.68 [0.3, 1.54]	0.354
New AF	30 (32.26)	12 (26.09)	18 (38.30)	0.268	0.57 [0.24, 1.37]	0.210
Depression	7 (7.53)	2 (4.35)	5 (10.64)	0.435	0.38 [0.07, 2.08]	0.265
History of Stroke/TIA	19 (20.43)	9 (19.57)	10 (21.28)	1.000	0.9 [0.33, 2.47]	0.838

Figures in parentheses are percentages. *P < 0.05 as the threshold for statistical significance. P values are uncorrected for multiple comparisons.

Abbreviations: LCVF = Late venous phase cortical vein filling; OTT = Onset to treatment time (time to tPA); AF = Atrial fibrillation; TIA = Transient Ischemic Attack; IQR = Inter-quartile range; SD = Standard deviation; NIHSS = National Institute of Health Stroke Scale Score.

**Table 2:** List of short term and long term clinical outcomes stratified by late-stage cortical vein filling.

	All patients	Delayed-LCVF	Non-delayed-LCVF	P	OR [95% CI]	P > |z|
	(n = 93)	(n = 46)	(n = 47)			
NIHSS at 24 hours; Median [IQR]	7 [9]	7.5 [12]	5 [10]	0.111	1.03 [0.97, 1.09]	0.348
24-Hour Core Volume (in mL); median [IQR]	15.8 [32.4]	18.05 [37.7]	12.5 [32.9]	0.3727	1 [1, 1.01]	0.247
Penumbral Salvage (in mL); median [IQR]	39.55 [79.25]	24.15 [84.4]	43.45 [77.4]	0.3100	1 [0.99, 1]	0.272
% Penumbral Salvage; Median [IQR]	65.55 [91.8]	52.92 [141.18]	84.24 [73.8]	0.1008	1 [0.997, 1]	0.189
Reperfusion Status at 24 hours				<0.001*		<0.001*
*Major Reperfusion*	49 (52.69)	16 (34.78)	33 (70.21)	0.001*	0.48 [0.17, 1.35]	0.167
*Partial Reperfusion*	22 (23.66)	19 (41.3)	3 (6.38)	<0.001*	6.33 [1.44, 27.73]	0.014*
*No Reperfusion*	22 (23.66)	11 (23.91)	11 (23.40)	1.0000	1 [ref]	
Reperfusion Status at 24 hours				0.001*		0.0008*
*Major Reperfusion*	49 (52.7)	16 (32.6)	33 (67.4)	0.001*	1 [ref]	
*Poor Reperfusion*	44 (47.3)	30 (68.2)	14 (31.8)	0.001*	4.42 [1.85, 10.56]	0.001*
mRS at 90 days; Median [IQR]	2 [3]	2 [3]	2 [4]	0.1424		
*Good (mRS* = *0–2)*	54 (58.06)	24 (52.17)	30 (63.83)	0.297	0.62 [0.27, 1.42]	0.256
*Bad (mRS* = *3–6)*	39 (41.94)	22 (47.83)	17 (36.17)	0.297	1.61 [0.71, 3.71]	0.256

Figures in parentheses are percentages and those in square brackets are interquartile ranges. *P < 0.05 as the threshold for statistical significance. Comparison between two groups for categorical and continuous/ordinal variables was made using 2-sided Fisher’s exact test and Wilcoxon rank-sum (Mann-Whitney) test, respectively. Abbreviations: mRS = Modified Rankin Score. P values shown are uncorrected for multiple comparisons.

**Table 3:** Multivariate regression model for associations with proximal clot location.

Model 3A			
*Final model: First Step*	OR	95% CI	P > |z|
*Model characteristics: n* = *93; dof* = *6; AIC* = *88.57; BIC* = *103.77; Sensitivity* = *82.05*%; *Specificity* = *81.48*%; *PPV* = *76.19*%; *NPV* = *86.27*%; *Overall rate of classification* = *81.72*%; *ROC area* = *0.8882; Pseudo R2* = *0.3947*			
Delayed-LCVF	110.33	11.92, 1020.97	<0.0001*
TPME	0.56	0.34, 0.91	0.020*
Dyslipidaemia	5.89	1.59, 21.82	0.008*
Poor collateral	1.05	0.25, 4.36	**0.949**
Reperfusion status			**0.8382**
*Poor reperfusion*	0.88	0.27, 2.93	**0.838**
*Major Reperfusion*	1 [Ref]		
**Model 3B**			
***Reduced model without delayed-LCVF***	OR	95% CI	P > |z|
*Model characteristics: n* = *93; dof* = *3; AIC* = *123.6; BIC* = *131.24; Sensitivity* = *35.9*%; *Specificity* = *81.48*%; *PPV* = *58.33*%; *NPV* = *63.77*%; *Overall rate of classification* = *62.37*%; *ROC area* = *0.6624; Pseudo R2* = *0.07*			
TPME	1.31	0.99, 1.72	0.057
Dyslipidaemia	2.62	1.1, 6.26	0.03*
**Model 3C**			
***Reduced model with delayed-LCVF***:***Last Step***	OR	95% CI	P > |z|
*Model characteristics: n* = *93; dof* = *4; AIC* = *84.62; BIC* = *94.75; Sensitivity* = *82.05*%; *Specificity* = *81.48*%; *PPV* = *76.19*%; *NPV* = *86.27*%; *Overall rate of classification* = *81.72*%; *ROC area* = *0.8865; Pseudo R2* = *0.3943*			
Delayed-LCVF	106.62	15.04, 755.76	<0.0001*
TPME	0.56	0.35, 0.92	0.021*
Dyslipidaemia	5.84	1.62, 21.03	0.007*

P < 0.1 for enter and stay criteria. The two multivariate models differed in terms of the particular covariates/factors included. *P < 0.05 as the threshold for statistical significance.

Abbreviations: dof = degrees of freedom; OR = Odds Ratio; AIC and BIC = Akaike and Bayesian information criteria; PPV = Positive predictive value; NPV = Negative predictive value; ROC: Receiver-operator characteristic curve; CI = Confidence interval; TPME = Time to peak of maximum arterial enhancement (in seconds); LCVF = Late venous phase cortical vein filling; NIHSS = National Institute of Health Stroke Scale Score.

**Table 4:** Stepwise backwards multivariate regression modelling for associations with 24 hours’ poor angiographic reperfusion.

*Model 4A*			
*Final model: First Step*	Mixed OR	95% CI	P > |z|
*Model characteristics: n* = *92; dof* = *8; AIC* = *119.775; BIC* = *139.95; Sensitivity* = *75*%; *Specificity* = *66.67*%; *PPV* = *67.35*%; *NPV* = *74.42*%; *Overall rate of classification* = *70.65*%; *ROC area* = *0.7704; Pseudo R2* = *0.1852*			
Delayed-LCVF	2.26	0.58, 8.73	0.238
NIHSS at admission	1.03	0.95, 1.12	0.490
Age	1.03	0.99, 1.08	0.184
Acute Core volume (in mL)	1.02	1, 1.04	0.037*
Poor Collaterals	1.95	0.56, 6.82	0.296
Hypertension	0.52	0.11, 2.47	0.408
Clot location			0.6264
*Proximal thrombus*	1.33	0.42, 4.24	0.626
*Distal thrombus*	1 [Ref]		
***Model 4B***			
***Reduced Model without delayed-LCVF***	Mixed OR	95% CI	P > |z|
*Model characteristics: n* = *92; dof* = *3; AIC* = *120.64; BIC* = *128.21; Sensitivity* = *70.45*%; *Specificity* = *64.58*%; *PPV* = *64.58*%; *NPV* = *70.45*%; *Overall rate of classification* = *67.39*%; *ROC area* = *0.7015; Pseudo R2* = *0.0999*			
Clot location			0.0174*
*Proximal thrombus*	2.94	1.21, 7.17	0.017*
*Distal thrombus*	1 [Ref]		
Acute Core volume (in mL)	1.02	1.004, 1.038	0.015*
***Model 4C***			
***Reduced model with delayed-LCVF***:***Last Step***	Mixed OR	95% CI	P > |z|
*Model characteristics: n* = *92; dof* = *4; AIC* = *117.05; BIC* = *127.13; Sensitivity* = *70.45*%; *Specificity* = *68.75*%; *PPV* = *67.39*%; *NPV* = *71.74*%; *Overall rate of classification* = *69.57*%; *ROC area* = *0.7353; Pseudo R2* = *0.1438*			
Delayed-LCVF	3.72	1.22, 11.28	0.021*
Clot location			0.5729
*Proximal thrombus*	1.38	0.45, 4.21	0.573
*Distal thrombus*	1 [Ref]		
Acute Core volume (in mL)	1.02	1.0032, 1.04	0.021*

P < 0.1 for enter and stay criteria. The two multivariate models differed in terms of the particular covariates/factors included. *P < 0.05 as the threshold for statistical significance.

Abbreviations: dof = degrees of freedom; OR = Odds Ratio; AIC and BIC = Akaike and Bayesian information criteria; PPV = Positive predictive value; NPV = Negative predictive value; ROC: Receiver-operator characteristic curve; CI = Confidence interval; LCVF = Late venous phase cortical vein filling; NIHSS = National Institute of Health Stroke Scale Score.

**Table 5:** Stepwise backwards multivariate regression modelling for associations with 90 days’ bad functional outcome.

*Model 5A*			
*Final model: First Step*	Mixed OR	95% CI	P > |z|
*Model characteristics: n* = *92; dof* = *8; AIC* = *109.27; BIC* = *129.44; Sensitivity* = *71.79*%; *Specificity* = *79.25*%; *PPV* = *71.79*%; *NPV* = *79.25*%; *Overall rate of classification* = *76.09*%; *ROC area* = *0.8036; Pseudo R2* = *0.2562*			
Delayed-LCVF	0.91	0.24, 3.38	0.884
NIHSS at admission	1.02	0.93, 1.13	**0.624**
Acute Core volume (in mL)	1.02	0.999, 1.05	**0.058**
Penumbra volume (in mL)	0.999	0.99, 1.01	**0.969**
Clot location			**0.4991**
*Proximal thrombus*	0.64	0.17, 2.37	0.499
*Distal thrombus*	1 [Ref]		
Reperfusion status			0.0001*
*Poor reperfusion*	9.05	2.92, 28.05	<0.0001*
*Major reperfusion*	1 [Ref]		
Dyslipidaemia	1.03	0.35, 2.97	0.961
***Model 5B***			
***Reduced Model without delayed-LCVF***	Mixed OR	95% CI	P > |z|
*Model characteristics: n* = *92; dof* = *5; AIC* = *103.2977; BIC* = *115.91; Sensitivity* = *69.23*%; *Specificity* = *79.25*%; *PPV* = *71.05*%; *NPV* = *77.78*%; *Overall rate of classification* = *75*%; *ROC area* = *0.8021; Pseudo R2* = *0.2560*			
NIHSS at admission	1.02	0.93, 1.12	0.612
Acute Core volume (in mL)	1.02	0.999, 1.046	0.051
Clot location			0.3677
*Proximal thrombus*	0.61	0.21, 1.78	**0.368**
*Distal thrombus*	1 [Ref]		
Reperfusion status			0.0001*
*Poor reperfusion*	8.82	3.01, 25.86	<0.0001*
*Major reperfusion*			
***Model 5C***			
***Reduced model with delayed-LCVF***:***Last Step***	Mixed OR	95% CI	P > |z|
*Model characteristics: n* = *92; dof* = *7; AIC* = *74.83; BIC* = *92.49; Sensitivity* = *82.05*%; *Specificity* = *88.68*%; *PPV* = *84.21*%; *NPV* = *87.04*%; *Overall rate of classification* = *85.87*%; *ROC area* = *0.93; Pseudo R2* = *0.5149*			
Delayed-LCVF	0.90	0.25, 3.27	0.875
NIHSS at admission	1.02	0.93, 1.12	0.618
Acute Core volume (in mL)	1.02	0.999, 1.05	**0.051**
Clot location			0.4891
*Proximal thrombus*	0.64	0.18, 2.24	0.489
*Distal thrombus*	1 [Ref]		
Reperfusion status			0.0001*
*Poor reperfusion*	9.05	2.94, 27.83	<0.0001*
*Major reperfusion*	1 [Ref]		

^1^Used as reference category.

P < 0.1 for enter and stay criteria. The two multivariate models differed in terms of the particular covariates/factors included. *P < 0.05 as the threshold for statistical significance.

Abbreviations: dof = degrees of freedom; OR = Odds Ratio; AIC and BIC = Akaike and Bayesian information criteria; PPV = Positive predictive value; NPV = Negative predictive value; ROC: Receiver-operator characteristic curve; CI = Confidence interval; TPME =Time to peak of maximum arterial enhancement (in seconds); LCVF = Late venous phase cortical vein filling; NIHSS = National Institute of Health Stroke Scale Score.
