# Supplementary material for: Association of Cortical Vein Filling with Clot Location and Clinical Outcomes in Acute Ischaemic Stroke Patients
Source: Sci Rep. 2016 Dec 5;6:38525. doi: 10.1038/srep38525 (PMC5137111; doi:10.1038/srep38525)
Supplement: Supplementary Information [file srep38525-s1.doc]

**SUPPLEMENTARY INFORMATION (SI)**

**Association of cortical vein filling with clot location and clinical outcomes in acute ischaemic stroke patients**

Sonu Bhaskar, Andrew Bivard, Peter Stanwell, John R Attia, Mark Parsons, Michael Nilsson, and Christopher Levi

**Supplementary Table 1. Demographic and baseline characteristics by the outcome (mRS) at 3 months after intravenous thrombolysis. Odds ratio (OR) were obtained by bivariate logistic regression analysis for association with good functional outcome (mRS=0-2).**

| Characteristics | Good Outcome;  N (%)=54(58.06) | Bad outcome;  N (%)= 39(41.94) | P | OR (95% CI) | P>|z| |
| --- | --- | --- | --- | --- | --- |
| Age; Mean±SD | 70±18 | 76±14 | 0.118 | 0.97 [0.94, 1.01] | 0.117 |
| NIHSS at admission; Median [IQR] | 12 [8] | 15 [8] | 0.057 | 0.92 [0.86, 1.00] | 0.039* |
| NIHSS at 24 hours; Median [IQR] | 4 [4] | 13 [9] | <0.001* | 0.73 [0.64, 0.83] | <0.001* |
| OTT (in mins); Mean±SD | 152.28±74.71 | 176.77±94.29 | 0.2090 | 1 [0.99, 1] | 0.172 |
| Acute Core Volume; Median [IQR] | 10 [20.2] | 20.4 [55.5] | 0.003* | 0.97 [0.95, 0.99] | 0.004* |
| Penumbra; Median [IQR] | 51.9 [86.2] | 71.8 [60.3] | 0.081 | 0.99 [0.98, 1] | 0.086 |
| TPME; Median [IQR] | 8 [2] | 8 [2] | 0.847 | 1.08 [0.84, 1.38] | 0.567 |
| Delayed-LCVF | 24 (44.44) | 22 (56.41) | 0.297 | 0.62 [0.27, 1.42] | 0.256 |
| Female; n (%) | 27 (50) | 22 (56.41) | 0.674 | 0.77 [0.34, 1.77] | 0.542 |
| Good Collaterals; n (%) | 28 (51.85) | 16 (41.03) | 0.400 | 1.55 [0.67, 3.56] | 0.303 |
| Poor Collaterals; n (%) | 26 (48.15) | 23 (58.97) | 0.400 | 0.65 [0.28, 1.48] | 0.303 |
| Clot location |  |  | 0.191 |  | 0.2016 |
| Proximal thrombus  (ICA + M1P) | 22 (40.74) | 17 (43.59) | 0.833 | 0.89 [0.39, 2.04] | 0.784 |
| Distal thrombus  (M1D + M2 + M3) | 32 (59.26) | 22 (56.41) | 0.833 | 1 [Ref] |  |
|  |  |  |  |  |  |
| *ICA* | 12 (22.22) | 6 (15.38) | 0.441 | 0.86 [0.24, 3] | 0.809 |
| *M1 Proximal (M1P)* | 10 (18.52) | 11 (28.21) | 0.319 | 0.39 [0.12, 1.24] | 0.111 |
| *M1 Distal (M1D)* | 11 (20.37) | 13 (33.33) | 0.230 | 0.36 [0.12, 1.11] | 0.076 |
| *M2 and M3* | 21 (38.89) | 9 (23.08) | 0.122 | 1 [ref] |  |
|  |  |  |  |  |  |
| 24-Hour Core Volume (in mL); median [IQR] | 16.1 [32.4] | 15.8 [35] | 0.5911 | 1 [1, 1.01] | 0.323 |
| Reperfusion Status |  |  |  |  | <0.0001* |
| *Major Reperfusion* | 40 (74.07) | 9 (23.08) | <0.0001* | 1 [ref] |  |
| *Poor Reperfusion* | 14 (25.93) | 30 (76.92) | <0.0001* | 0.11 [0.04, 0.27] | <0.0001* |
|  |  |  |  |  |  |
| Risk factors |  |  |  |  |  |
| *Hypertension* | 50 (92.59) | 32 (82.05) | 0.192 | 2.73 [0.74, 10.1] | 0.131 |
| *Diabetes* | 18 (33.33) | 9 (23.08) | 0.357 | 1.67 [0.65, 4.25] | 0.284 |
| *Dyslipidemia* | 23 (42.59) | 17 (43.59) | 1.000 | 2.59 [1.11, 6.05] | 0.028* |
| *Present Smoker* | 15 (27.78) | 10 (25.64) | 1.000 | 1.12 [0.44, 2.83] | 0.807 |
| *Past Smoker* | 23 (42.59) | 15 (38.46) | 0.831 | 1.46 [0.63, 3.37] | 0.378 |
| *AF* | 31 (57.41) | 20 (51.28) | 0.673 | 0.93 [0.41, 2.13] | 0.870 |
| *New AF* | 20 (37.04) | 10 (25.64) | 0.270 | 0.72 [0.3, 1.77] | 0.478 |
| *Depression* | 6 (11.11) | 1 (2.56) | 0.232 | 1.04 [0.22, 4.94] | 0.959 |
| *History of Stroke/TIA* | 10 (18.52) | 9 (23.08) | 0.612 | 0.77 [0.27, 2.16] | 0.615 |

**Supplementary Table 2.** Demographic and baseline characteristics of all patients and by the locus of the thrombus. Odds ratio (OR) were obtained by bivariate logistic regression analysis for association with proximal clot.

| Characteristics | **Proximal thrombus**  **(ICA + M1P)**  **(n=39)** | **Distal thrombus**  **(M1D + M2 + M3)**  **(n=54)** | P | **OR (95% CI)** | **P>|z|** |
| --- | --- | --- | --- | --- | --- |
| Age (in years); Mean±SD | 72±14 | 72.5±18 | 0.711 | 1.01 [0.98, 1.05] | 0.420 |
| Female; n (%) | 17 (43.59) | 32 (59.26) | 0.147 | 0.53 [0.23, 1.11] | 0.137 |
| **NIHSS at admission; Median [IQR]** | 14 [8] | 12.5 [8] | 0.184 | 1.04 [0.97, 1.11] | 0.300 |
| **NIHSS at 24 hours; Median [IQR]** | 7 [12] | 6.5 [10] | 0.343 | 1.01 [0.96, 1.07] | 0.682 |
| OTT (in mins); Mean±SD | 159.49±67.65 | 164.76±94.44 | 0.6369 | 1 [0.99, 1] | 0.764 |
| Acute Core Volume; Median [IQR] | 16.3 [25] | 12.8 [27.1] | 0.884 | 1.0 [0.99, 1.01] | 0.847 |
| Penumbra; Median [IQR] | 60 [70.8] | 66.8 [77.9] | 0.391 | 1.0 [0.99, 1.01] | 0.384 |
|  |  |  |  |  |  |
| TPME; Median [IQR] | 8 [4] | 8 [2] | **0.019*** | 1.3 [1, 1.71] | 0.054 |
| Delayed-LCVF | 33 (84.62) | 13 (24.07) | **<0.001*** | 17.35 [5.95, 50.59] | **<0.001*** |
| **Baseline Collateral Status** |  |  | **<0.001*** |  |  |
| Good Collaterals; n (%) | 9 (23.08) | 35 (64.81) | **<0.001*** | 0.16 [0.06, 0.41] | **<0.001*** |
| Poor Collaterals; n (%) | 30 (76.92) | 19 (35.19) | **<0.001*** | 6.14 [2.42, 15.58] | **<0.001*** |
| **Reperfusion Status** |  |  |  |  | **0.0209*** |
| *Major Reperfusion* | 15 (38.46) | 34 (62.96) | 0.022* | 1 [Ref] |  |
| *Poor Reperfusion* | 24 (61.54) | 20 (37.04) | 0.022* | 2.72 [1.16, 6.36] | **0.021*** |
| 24-Hour Core Volume (in mL); median [IQR] | 11.2 [23.8] | 18 [38] | 0.3874 | 1 [0.99, 1] | 0.339 |
| **mRS at 90 days; Median [IQR]** | 2 [3] | 2 [4] | 0.803 |  |  |
| **Good (mRS= 0-2)** | 22 (56.41) | 32 (59.26) | 0.833 | 0.89 [0.39, 2.05] | 0.784 |
| **Bad (mRS= 3-6)** | 17 (43.59) | 22 (40.74) | 0.833 | 1.12 [0.49, 2.59] | 0.784 |
| **Risk factors** |  |  |  |  |  |
| *Hypertension* | 32 (82.05) | 50 (92.59) | 0.192 | 0.37 [0.1, 1.4] | 0.131 |
| *Diabetes* | 12 (30.77) | 15 (27.78) | 0.819 | 1.16 [0.47, 2.85] | 0.754 |
| *Dyslipidemia* | 22 (56.41) | 18 (33.33) | **0.034*** | 2.59 [1.11, 6.05] | **0.028*** |
| *Present Smoker* | 11 (28.21) | 14 (25.93) | 0.817 | 1.12 [0.44, 2.83] | 0.807 |
| *Past Smoker* | 18 (46.15) | 20 (37.04) | 0.400 | 1.46 [0.63, 3.37] | 0.378 |
| *AF* | 21 (53.85) | 30 (55.56) | 1.000 | 0.93 [0.41, 2.13] | 0.870 |
| *New AF* | 11 (28.21) | 19 (35.19) | 0.509 | 0.72 [0.3, 1.77] | 0.478 |
| *Depression* | 3 (7.69) | 4 (7.41) | 1.000 | 1.04 [0.22, 4.94] | 0.959 |
| *History of Stroke/TIA* | 7 (17.95) | 12 (22.22) | 0.795 | 0.77 [0.27, 2.16] | 0.615 |

**Supplementary Table 3. Univariate logistic regression analysis for 24 hours’ poor angiographic reperfusion. *p<0.05 as threshold for statistical significance**

| Characteristics | **Poor angiographic reperfusion** | **Major angiographic reperfusion** | **P** | **OR (95% CI)** | **P>|z|** |
| --- | --- | --- | --- | --- | --- |
| Age (in years); Mean±SD | 74.16±10.34 | 69.31±13.68 | **0.067** | 1.03 [0.998, 1.07] | **0.064** |
| Female; n (%) | 23 (52.27) | 26 (53.06) | 1.000 | 0.97 [0.43, 2.19] | 0.939 |
| **NIHSS at admission; Median [IQR]** | 14.5 [8] | 13 [9] | **0.096** | 1.08 [1, 1.16] | **0.05*** |
| **NIHSS at 24 hours; Median [IQR]** | 11.5 [11.5] | 4 [5] | **<0.00001*** | 1.21 [1.11, 1.33] | **<0.0001*** |
| OTT (in mins); Mean±SD | 166.93±88.01 | 158.61±80.69 | 0.7695 | 1.001 [0.996, 1.006] | 0.632 |
| Acute Core Volume; Median [IQR] | 20.35 [54.15] | 9.9 [19.25] | **0.0639** | 1.02 [1.003, 1.04] | **0.023*** |
| Penumbra; Median [IQR] | 73.5 [61.95] | 54.65 [78.8] | 0.1152 | 1.007 [0.998, 1.02] | 0.11 |
| **Baseline Collateral Status** |  |  |  |  |  |
| Good Collaterals; n (%) | 14 (31.82) | 30 (61.22) | **0.007*** | 0.296 [0.13, 0.696] | **0.005*** |
| Poor Collaterals; n (%) | 30 (68.18) | 19 (38.78) | **0.007*** | 3.38 [1.44, 7.96] | **0.005*** |
|  |  |  |  |  |  |
| TPME; Median [IQR] | 8 [2] | 8 [2] | 0.1584 | 1.15 [0.89, 1.47] | 0.289 |
| Delayed-LCVF | 30 (68.18) | 16 (32.65) | **0.001*** | 4.42 [1.85, 10.56] | **0.001*** |
| Clot location |  |  |  |  | **0.0209*** |
| Proximal thrombus  (ICA + M1P) | 24 (54.55) | 15 (30.61) | **0.022*** | 2.72 [1.16, 6.36] | **0.021*** |
| Distal thrombus  (M1D + M2 + M3) | 20 (45.45) | 34 (69.39) | **0.022*** | [Ref] |  |
| 24-Hour Core Volume (in mL); median [IQR] | 13.65 [30.95] | 17.1 [32.9] | 0.4813 | 0.996 [0.99, 1.004] | 0.384 |
| **mRS at 90 days; Median [IQR]** | 3 [3] | 1 [2] | **<0.00001*** |  |  |
| **Good (mRS= 0-2)** | 14 (31.82) | 40 (81.63) | **<0.0001*** | 0.11 [0.04, 0.27] | **<0.0001*** |
| **Bad (mRS= 3-6)** | 30 (68.18) | 9 (18.37) | **<0.0001*** | 9.52 [3.64, 24.92] | **<0.0001*** |
| **Risk factors** |  |  |  |  |  |
| *Hypertension* | 36 (81.82) | 46 (93.88) | **0.108** | 0.29 [0.07, 1.19] | **0.085** |
| *Diabetes* | 11 (25) | 16 (32.65) | 0.495 | 0.69 [0.28, 1.7] | 0.418 |
| *Dyslipidemia* | 20 (45.45) | 20 (40.82) | 0.680 | 1.21 [0.53, 2.75] | 0.652 |
| *Present Smoker* | 12 (27.27) | 13 (26.53) | 1.000 | 1.04 [0.41, 2.6] | 0.936 |
| *Past Smoker* | 19 (43.18) | 19 (38.78) | 0.679 | 1.2 [0.52, 2.75] | 0.666 |
| *AF* | 24 (54.55) | 27 (55.10) | 1.000 | 0.98 [0.43, 2.22] | 0.957 |
| *New AF* | 12 (27.27) | 18 (36.73) | 0.379 | 0.65 [0.27, 1.56] | 0.331 |
| *Depression* | 2 (4.55) | 5 (10.2) | 0.44 | 0.42 [0.08, 2.28] | 0.314 |
| *History of Stroke/TIA* | 9 (20.45) | 10 (20.41) | 1.000 | 1 [0.37, 2.75] | 0.996 |
